# Sensitive Dentine in a Pulpless Tooth

**Published:** 1866-02

**Authors:** Henry S. Chase

**Affiliations:** Iowa City, Iowa


### SENSITIVE DENTINE IN A PULPLESS TOOTH.

By Henry S. Chase m.d.

### A CASE.

Mrs. R., age 30. Nervous temperament, blonde, called in May last for cure of periostitis, and fistulous discharge from right superior first molar. Had been plugged two years before. The cavity being very sensitive, her dentist had used arsenic to destroy sensitiveness of dentine. Was then filled without pain. Some weeks after the operation it had ached, and finally discharged matter through the gum. Had "ulcerated" every few weeks since that time.

I removed the plug and found a superficial cavity on the anterior proximal surface very little deeper than the thickness of the enamel. Drilled to the pulp cavity, enlarged it, syringed, and forced creosote through fistula. One week after, treated with creosote again. July 8th, plugged with Hill's stopping, as there had been no discharge since last injection. Previously had a temporary plug of cotton and sandarac.

Dec. 9th. Has been a little "sore" lately, but no discharge. Removed plug, syringed, and wiped with creosote. Was much surprised to find the dentine of the pulp cavity very painful to the touch of a naked instrument; even wiping with cotton and creosote causes pain. No pain on lingual side of cavity or in the lingual root. Probed the roots to see if there were any living vessels left in them. Could not cause pain by forcing air or fluids in. The fistulous opening was on the outside of the jaw.

Dec. 13th. Tooth comfortable in the socket and no sensitiveness in pulp cavity. Plugged with tin foil.

I think this is the only case I ever observed of the kind. What caused this sensibility? I think it must have proceeded from pressure of the contents of the dental tubuli on the inflamed alveolo-dental periosteum. Or the sensation may have been caused by open dental tubes impinging on nervous filaments accompanying blood vessels in the cells of the cementum.

This case illustrates the danger of using arsenic in even *superficial* cavities, where it is *not* desired to destroy the pulp.

*Iowa City, Iowa.*

